# Exploring neurology resident experiences with a no-prep journal club to learn research study design and critical appraisal

**DOI:** 10.1186/s12909-025-08275-4

**Published:** 2025-12-29

**Authors:** Katherine A. Fu, Sally Elting, Joy M. Chan, Roy E. Strowd

**Affiliations:** 1 Department of Neurology, University of California, Los Angeles, CA USA; 2 Department of Neurology, Wake Forest University, Winston-Salem, NC USA

**Keywords:** Journal club, Critical appraisal, Research study design, Graduate medical education, Evidence-based medicine, Qualitative methods

## Abstract

**Background:**

Critical appraisal of research is an important skill in practicing evidence-based medicine. Traditional journal clubs often involve pre-session preparation, which can be challenging for resident physicians with competing professional commitments. To address these challenges, the UCLA neurology residency program implemented a "no-prep" journal club format, eliminating prerequisite preparation and emphasizing active, collaborative learning. This study uses a qualitative approach within a constructivist paradigm to explore neurology residents' experiences and the influence of this format on learning critical appraisal.

**Methods:**

We conducted four monthly no-prep journal clubs during the 2023–2024 academic year. A qualitative study design was used, and data were collected via one-on-one semi-structured interviews with neurology residents. Interview transcripts were descriptively coded, categorized, and analyzed to identify themes using inductive thematic analysis.

**Results:**

Nine neurology residents were interviewed, and five themes were identified. Participants felt that the no-prep journal club format established a psychologically safe learning environment, as it removed participation hesitancy due to inadequate preparation. This format helped learners identify their knowledge gaps, situating them in their zones of proximal development. The presence of the resident and faculty facilitators offered scaffolding for residents to navigate beyond this zone toward independent study design. Regarding behavior change, residents mentioned the format promoted active critique when reading research and the application of this information to patient care. Bookending the session with a clinical case promoted engagement by grounding the format in clinical relevance.

**Conclusions:**

This no-prep journal club format reflected core elements of constructivist theory. Residents’ prompt identification of knowledge gaps illustrated Vygotsky’s zone of proximal development, while facilitator support provided the scaffolding necessary to advance their learning. Traditional and flipped classroom style journal club formats are challenged by the necessary prerequisite preparation. In contrast, the no-prep journal club illustrates how learning efficiency in critical appraisal can be improved even in the absence of preparatory work.

**Supplementary Information:**

The online version contains supplementary material available at 10.1186/s12909-025-08275-4.

## Background

Residents often participate in journal clubs to learn the skills involved in critical appraisal of research, yet competing professional commitments lead to inadequate preparation, which limits their learning during these sessions. Journal clubs are a common way to teach critical appraisal of research to physicians, dating back to Osler [1]. The traditional journal club format generally involves a resident and/or faculty facilitator presenting a research article to a group, inviting questions and discussion either throughout the session or towards the end [2]. At UCLA, neurology resident journal clubs are held once per month during the Wednesday morning academic half day for a total of 4–6 sessions per year. There is a 75% attendance requirement for neurology resident didactics. Sessions typically occur during lunchtime and had historically implemented the traditional journal club format through 2023. Challenges identified in this format include low attendance and lack of participation [3]. To improve engagement and learning during journal club, others have explored alternatives to this traditional format including the use of interactive formats, video conferencing platforms, and social media [4–6]. A previous study comparing an active learning, flipped classroom-style journal club format with a traditional format noted improvements in neurology resident learning outcomes. Outcomes pertaining to clinical application of research, but not research methodology, improved with the active learning format. However, the active learning format demonstrated disadvantages such as the burden of pre-work and an artificial nature of dialogue generated during these sessions [3].

An ideal journal club format would therefore alleviate the demands of preparation, promote active engagement, and improve learning outcomes regarding research methodology and application to patient care. Without further exploration of such a format, we risk future healthcare professionals becoming outpaced by the large volumes of information and rapid research advances present in the current era, struggling to practice evidence-based medicine (EBM). The “no-prep” approach encourages residents to design a research study in small groups based on the given background and research question of the article without pre-work [7]. This format was well-received by residents who expressed that the interactive nature and lack of preparatory work needed were advantages of this format [7]. However, how the no-prep format influences learning of critical appraisal skills remains unexplored.

We approached this study using a constructivist paradigm [8]. Constructivism asserts that learning occurs through building upon prior knowledge, integrating new information, and expanding upon previous experiences and understanding [9]. In a no-prep journal club format, neurology residents collaborate in small groups and use prior knowledge of study design and research methodology to propose a study after being given the research question and background. In this qualitative study of neurology residents, we aim to explore how this journal club format grounded in active learning strategies and a constructivist paradigm facilitates learning of the principles of study design and critical appraisal, defined by an understanding of research methodology and the clinical application of research.

## Methods

### Description of the “No-Prep” Journal club curriculum

**Fig. 1:**
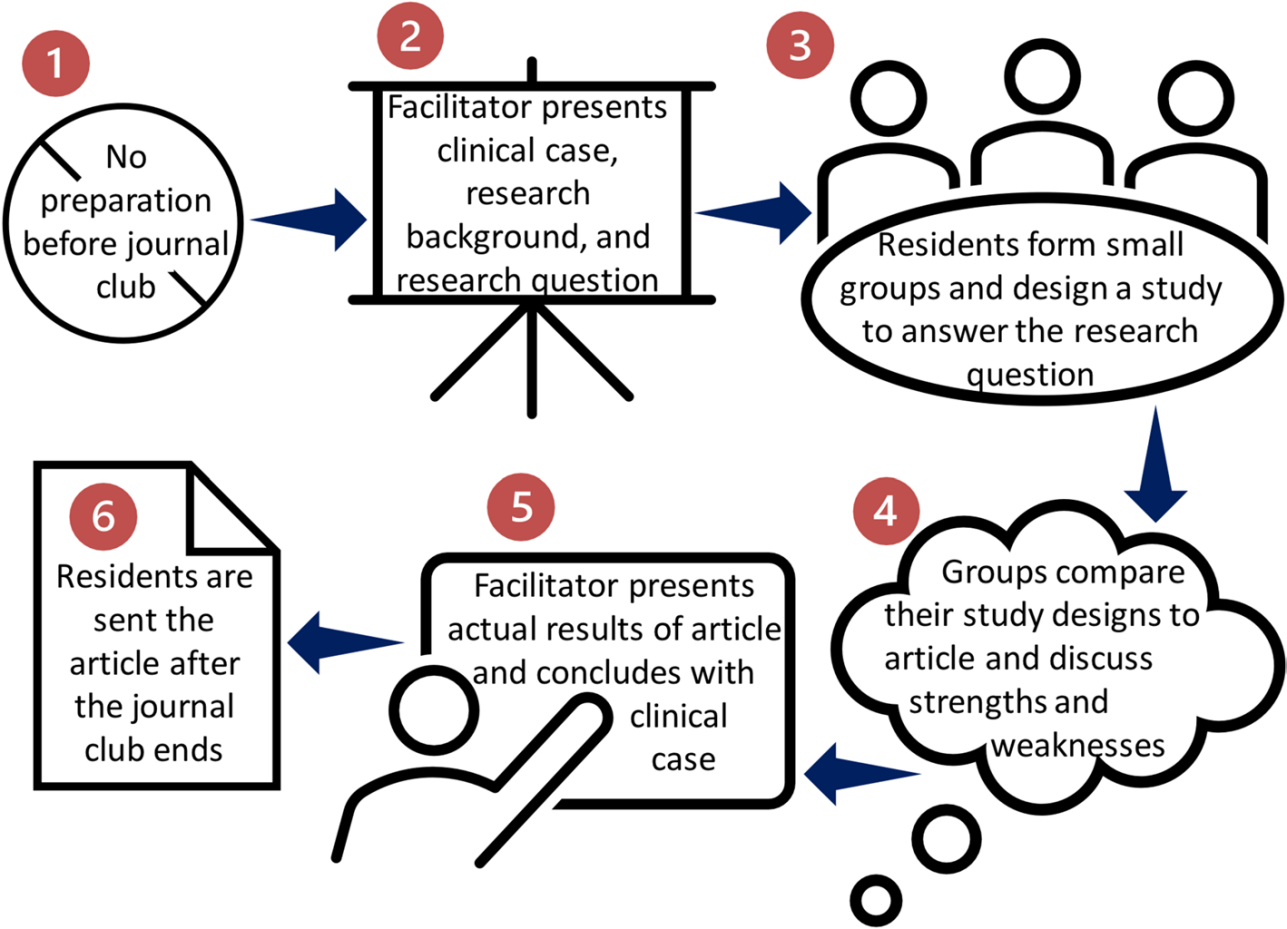
No-prep journal club format. Legend: Resident facilitators were instructed to allot a certain amount of time for each activity within the hour-long journal club session

The “no-prep” journal club curriculum was implemented with one journal club per month from January to April 2024 for a total of four one-hour long journal club sessions. Learning objectives for the curriculum can be found in Table 1. The format of the journal club was adapted from its implementation in the pediatrics literature and based on results from prior work (Fig. 1) [3, 7]. The resident facilitator opened with a clinical case, provided background on the research topic, posed the research question to the participants, and then asked neurology residents to design a study to best address the research question. Residents randomly divided into groups of approximately 3–5 to discuss their proposed study design. One resident facilitator and one faculty facilitator rotated through the groups during this time and provided additional guidance on how to structure or improve the proposed study design. At the conclusion of the small group discussion, small groups shared their proposed study designs with the larger group. Then, the resident facilitator shared the actual methods and learners compared their proposed study designs with that of the actual article. The resident facilitator proceeded to share the results of the study, and the session concluded by returning to the clinical case introduced in the beginning to discuss how the study’s results may influence their patient care approach.

**Table 1:** Learning objectives for the no-prep journal club curriculum

Learning Objectives
Recognize the key inclusion and exclusion criteria of participants enrolled in the discussed research study
Identify the relevant primary and secondary outcomes of the study
Discuss the strengths and weaknesses of the study design of the discussed journal club article and evaluate its appropriateness for the research question
Discuss the possible source of bias and limitations of the study
Determine the external validity (generalizability) of the results of the discussed clinical research article
Rate the importance of critical appraisal when analyzing research literature and applying this knowledge to patient care
Demonstrate proficiency when applying the findings of the discussed research article to patient care

### Qualitative approach

We used a qualitative study design with inductive thematic analysis as the methodological approach. Inductive thematic analysis is a flexible method for identifying, analyzing, and interpreting patterns of meaning within qualitative data, allowing themes to emerge directly from participants’ responses rather than from pre-existing theoretical frameworks [10]. This approach aligns well with the study’s exploration of how neurology residents experience an alternative journal club format by keeping an open mind to unanticipated insights and allowing patterns to emerge from the residents’ accounts.

### Context & sampling

Eligible neurology residents ranging from post-graduate year (PGY) 2 through 4 levels were invited to participate in 30-min semi-structured individual interviews using a purposive sampling approach based on participation in the journal club sessions. Invitations were sent by email with an attached information sheet. The email invitation was resent to residents who did not respond to the initial email. Thematic saturation was reached after interviewing nine residents. Participant demographics are displayed in Table 2.

**Table 2:** Interview participant demographics

Interview Participant Demographics	
Gender	Count
Male	4
Female	5
PGY Year	
2	4
3	2
4	3
Research Background (master’s or PhD)	
No	7
Yes	2

### Data collection

The interview protocol was created and revised by multiple researchers (SE, KF, RS). Questions pertained to the resident experience of the no-prep journal club and how this influenced their learning of research study design and critical appraisal of research. Data collection began in mid-September of 2024 and concluded mid-December of 2024.

One researcher (SE) conducted all semi-structured interviews via the Zoom virtual platform. The interviewer audio recorded interviews. These interviews were then transcribed prior to analysis.

### Data security & confidentiality

Interview files and transcripts were de-identified and labeled with a unique subject ID immediately after data collection and prior to analysis. Recordings were securely stored in encrypted digital formats on password-protected devices and servers. A password-protected file contained information linking the subject IDs to the participants’ email addresses.

### Data analysis

Interviews proceeded until thematic saturation was reached. Thematic saturation was assessed iteratively during data collection and analysis. As coding progressed, the research team tracked the emergence of new themes and considered data saturation achieved once redundancy was observed across participants and new themes were no longer being identified [11, 12].

Qualitative analysis was completed by two study investigators (SE and KF) using the coding software Dedoose (version 9.0.107; SocioCultural Research Consultants, LLC, Los Angeles, Calif). Initial codes were generated independently by two researchers (SE and KF) from two interview transcripts. Meaning units from these transcripts were identified, condensed, and coded. After reaching consensus over several meetings with both investigators, the codebook was generated. Inter-coding reliability was achieved with a Cohen’s Kappa coefficient of 0.85, calculated using the Dedoose software [13]. This codebook was then applied to the remaining transcripts and the codes were grouped into categories. Themes were developed from these categories in order to capture latent content. Themes were iteratively revised until consensus was reached. To increase transparency, we provide examples of how meaning units were condensed, coded, categorized, and turned into themes in Table 3.

**Table 3:** Data analysis process

Meaning Unit (Participant Quote)	Condensed Meaning Unit	Code	Category	Themes
*“I don't think it's realistic to expect people to always be able to read a whole journal article and come forward with notes ready to start so by eliminating that pressure to study in advance, you really encourage participation.” -R6*	Eliminating preparation pressure encourages participation	Engagement	Environment supports engagement	Environment of psychological safety
*“It mainly pointed out the fact that we were not aware of those previously validated primary and secondary outcomes. So that was helpful to realize that knowledge gap and then have some possibilities pointed out.” -R3*	Journal club exposed unawareness of validated outcomes and gaps in knowledge	Study design	Critical appraisal learning	Identification of knowledge gaps
*“It's good when the faculty facilitator asks probing questions to the audience that get them to think and discuss it in a constructive way as opposed to just more like a lecture format, which I know it should be that way anyway, but obviously not everyone does it.” -R4*	Facilitator probing promotes discussion and constructive thinking	Faculty facilitator	Facilitator guidance	Facilitator scaffolding
*“It was helpful learning how transferable some of the research can be and also how to pay attention to those inclusion exclusion criteria when you're trying to apply that same information to your patients. So that was really helpful learning to appraise the research”. -R8*	Learning to appraise research and apply it to patients	Behavior change	Application of research to clinical practice	Active critique and clinical application
*“I tend to remember things more if there's a patient story. As my goal is to be a physician. Research is great, but the goal of research for me is for it to be helpful in practice, so it made it easy to see, this is the perfect situation where I could apply this information. And then when you have another case in clinic, then you can think of what are the key features of this case that I can apply this research to?” -R8*	Patient cases help translate research into clinical practice and enhance learning	Clinical case	Use of clinical cases to enhance learning	Problem-centered approach

### Trustworthiness and rigor

Credibility was established by triangulation of data across observations and semi-structured interviews [14, 15]. Dependability of the data was ensured by the use of multiple coders [14]. To ensure trustworthiness of data, we implemented member checking, and transcripts were sent to participants for review of accuracy and additional comments [14, 16]. Researchers SE and KF continually engaged in progressive subjectivity by intentionally re-examining expectations and assumptions of the study as the thematic analysis progressed [17]. SE and KF debriefed following interviews and discussed preliminary themes that were emerging over the course of data collection. An audit trail was also created as part of the analysis process, offering a timeline of the process through which initial codes were refined, merged, or renamed as patterns emerged [14].

### Reflexivity

When considering reflexivity, researcher KF acknowledged the potential influence of her leadership role as the Associate Program Director in the residency program on the interpretation of the results. To mitigate this, researcher SE who has no education role in the UCLA Department of Neurology conducted the interviews, coded transcripts, and participated in the thematic analysis. The interpretation of themes was also reviewed with researcher RS who similarly does not have a role in the residency program or in the Department of Neurology at UCLA.

## Results

Five primary themes were identified based upon the thematic analysis, outlined in Table 4.

**Table 4:** Key themes identified

Themes	
Theme #1: Environment of psychological safety	*“It allowed for a larger group discussion, because there needed to be no-prep, so there's less of a barrier for entry for everyone. Everyone, and anyone who is in the room was able to participate. And that's not the case for a normal journal club.” -R1*
Theme #2: Identification of knowledge gaps	*“It exposed the things that I don't know because if I were to have prepared something in advance by myself, I probably would have looked up the things I didn't know. […] It helped me recognize things I didn't know on the spot and which I didn't have a chance to read about before the session. So, it kind of exposed my growth areas pretty quickly.” -R6*
Theme #3: Facilitator scaffolding	*“It's good when the faculty facilitator or the student facilitator ask probing questions to the audience that get them to think and discuss it in a constructive way as opposed to just more like a lecture format. […] It’s better to facilitate learning and discussion.” -R4*
Theme #4: Active critique and clinical application	*“Regarding the sessions on Alzheimer's disease-modifying therapy, it certainly gave me more useful information to convey to patients who are asking about those disease modifying therapies. I was able to tell them in more detail about the outcomes of the studies, and the pros and cons of the drug.” -R6*
Theme #5: Problem-centered approach	*“From a clinician standpoint that is really helpful because it also makes you think, all right, well, now we have a patient who can participate in this trial or maybe cannot or shouldn't, even though some of the things qualify them. It really helps us prepare for our career, essentially. We're going to have updated studies and data, how do you interpret that and apply that to your practice?.” -R5*

### Theme 1: Environment of psychological safety

The “no-prep” aspect of the journal club created a culture of psychological safety that promoted discussion and engagement during the sessions. Multiple participants described an environment that facilitated participation because there was no expectation for learners to have prepared for the session beforehand.

*“It allowed for a larger group discussion, because there needed to be no-prep, so there's less of a barrier for entry for everyone. Everyone, and anyone who is in the room was able to participate. And that's not the case for a normal journal club.” -R1*

*“I feel like if we all got the journal, like got the paper a day or two ago, when you walk into the session, I would probably be a little bit more stressed going like, oh, we were expected to read this. And then if I didn't, then I would be like, oh my god, what am I doing here? But if you go in knowing that you're not supposed to know anything, it's a little bit more chill.” -R7*

*“It enhances learning in the sense that you don't assume that you read the paper beforehand. So if you assume that people will read the paper beforehand and you don't present enough data or background at the day of the journal club, then they're not going to get anything out of it.” -R4*

Neurology residents used language such as “less daunting”, “more chill”, “took a weight off my shoulders”, and “lower barrier to entry” among other words to describe the type of learning environment that the no-prep journal club fostered. Residents mentioned that in a traditional journal club or in an alternative format with an expectation for preparation, residents have varying amounts of time to read and prepare for the journal club session. This variability then leads to a perceived imbalance – that those who were on busy inpatient services and unable to adequately prepare felt disadvantaged and thus less likely to engage in the discussion. Residents acknowledged this perception of stress and the feeling that they should have prepared but felt conflicted in the setting of having limited time to do so. This then raises the possibility that if unfamiliar concepts were mentioned, these may have been attributed to a lack of preparation and would persist as a gap in knowledge as they were reluctant to ask questions or engage.

However, in this no-prep journal club, given that no one was afforded this benefit of preparation, there was the perception of a more psychologically safe and supportive environment equally accessible for all participants, engendering a more organic discussion.

### Theme 2: Identification of knowledge gaps

Residents applied their pre-existing knowledge of research methods, study design, and statistical analysis when engaging in the no-prep journal club. Many participants referenced prior coursework, clinical experience, and familiarity with concepts like randomized control trials, inclusion/exclusion criteria, and data analysis techniques. However, the challenge of actively designing a study exposed the limits of their understanding, as some struggled to apply their knowledge beyond familiar frameworks or recognized gaps in areas such as study methodology, statistical interpretation, and logistics or practical considerations in clinical trials design.

*“It exposed the things that I don't know because if I were to have prepared something in advance by myself, I probably would have looked up the things I didn't know. [...] It helped me recognize things I didn't know on the spot and which I didn't have a chance to read about before the session. So, it kind of exposed my growth areas pretty quickly.” -R6*

In contrast to the more passive approach of a traditional journal club, participants mentioned that the active learning and collaborative discussion components of the no-prep journal club session revealed potential areas of growth in their existing knowledge base.

### Theme 3: Facilitator scaffolding

Once these gaps in knowledge were identified, participants shared that the resident and faculty facilitators of the journal club were often key in their learning of study design and critical appraisal skills. Following the initial participant discussion, they would then often prompt for specific details and challenge participants to think critically about discrete elements of study design.

*“It's good when the faculty facilitator or the student facilitator ask probing questions to the audience that get them to think and discuss it in a constructive way as opposed to just more like a lecture format. […] It’s better to facilitate learning and discussion.” -R4*

*“It was good to have a subspecialist who's familiar with the condition, so Dr. X was there for the one that I was, so the amount of learning that we had, and also the context that we had about prior studies or similar studies would have been much less if he were not present.” -R3*

The resident and faculty facilitators with their expertise in the research methods of the actual study as well as their subspecialty expertise facilitated connections that helped learners transition from requiring guidance to achieving independence. Facilitators provided scaffolding for learners, which refers to the use of techniques to support the advancement of students toward independence [18], through their prompting questions and specific comments during both small and large group discussions. Examples of such comments included consideration of specific primary outcomes, the strengths and weaknesses of particular study designs, and examples of clinical application of the research results to patient care.

### Theme 4: Active critique and clinical application

Neurology residents mentioned that interactive discussions regarding critical appraisal and study design during journal club sessions promoted more active critique when reading research and the subsequent application of this information to patient care.

*“Regarding the sessions on Alzheimer's disease-modifying therapy, it certainly gave me more useful information to convey to patients who are asking about those disease modifying therapies. I was able to tell them in more detail about the outcomes of the studies, and the pros and cons of the drug.” -R6*

*“When reviewing data on, for example, NMO trials looking at how they define their primary endpoint and secondary endpoint, it's nice to be able to say, not just that a medication works in this type of patient, but it worked for specifically this outcome, maybe not these other outcomes, and so that would allow me to counsel patients in more detail about what exactly has been studied, what exactly I'm expecting, what we have less data on.” -R3*

Following the journal club curriculum, residents offered examples that described how their counseling of patients became more nuanced when interpreting research results. They were more mindful when focusing on specific elements of the research article and considering its clinical implications. For example, they recognized the relevance of the inclusion and exclusion criteria when counseling patients and considering the generalizability of study findings. As previously highlighted, others altered their patient discussions to focus more specifically on the primary outcome of a study and whether that outcome was applicable or meaningful to the care of their patient.

### Theme 5: Problem-centered approach

Neurology residents reported that they appreciated that the journal club session opened and closed with a clinical vignette, which made explicit the clinical application and relevance of the session and appealed to their professional identities as clinicians. The clinical case presentation appeals to the andragogical principle of a problem-centered approach to adult learning by contextualizing critical appraisal in real-world patient care and enhancing retention through practical application.

*“Having a patient case at the beginning and the end helps to contextualize the learning and make it clinically relevant and again, help people engage because not everybody's a research scientist, but everybody in the room is a clinician so it makes it relevant for everybody.” -R6*

*“From a clinician standpoint that is really helpful because it also makes you think, all right, well, now we have a patient who can participate in this trial or maybe cannot or shouldn't, even though some of the things qualify them. It really helps us prepare for our career, essentially. We're going to have updated studies and data, how do you interpret that and apply that to your practice?.” -R5*

Residents reported that the approach of framing the journal club session with a clinical case promoted the immediate relevance of the information presented during the session in a problem-centered manner. This relevance then enhanced the motivation to engage and participate during the session itself, as well as provided a concrete example for how the information learned could be applied to future clinical care.

## Discussion

This study explored resident experiences of a no-prep journal club and its influence on the learning of study design and critical appraisal. We used a qualitative approach situated in a constructivist paradigm, and the resulting themes were well-aligned with several principles of constructivism. In exploring resident experiences with this journal club format, our qualitative analysis revealed five primary themes.

Residents valued the importance of psychological safety in the learning environment, defined as the shared belief that individuals can express themselves without fear of embarrassment, judgment or negative consequences [19]. Within the no-prep journal club format, multiple participants described an atmosphere that encouraged participation, as there was no expectation for prior preparation. In contrast, traditional journal clubs rely on pre-session preparation, which can inadvertently create a hierarchy among participants based on their ability to allocate time for this pre-work [3]. This hierarchy may discourage individuals who have not had time to prepare from engaging in discussions, ultimately limiting opportunities for learning. Therefore, fostering psychological safety is foundational to any journal club format, as it ensures that all participants feel empowered to contribute, take intellectual risks, and actively engage in critical discussions.

Increased psychological safety combined with the challenge of designing a study set the stage for learners to identify gaps in their knowledge. The identification of knowledge gaps pushed participants into their Zone of Proximal Development (ZPD), which refers to the space between what a learner can do independently and what they can achieve with guidance from a more knowledgeable person [20]. According to Vygotsky, this is the space where the greatest learning potential exists. Unlike traditional journal clubs, where study design is often passively critiqued, the no-prep journal club format required participants to actively construct a study design before engaging with the actual methods of the provided research article. This process inherently revealed gaps in their understanding, as noted by participants who recognized limitations in their knowledge of study design in real-time. Situated within the broader theoretical framework of constructivism, ZPD underscores the idea that learning is not a passive process of knowledge transfer but an active, socially mediated experience in which individuals construct new understanding through interaction and engagement. Vygotsky’s social constructivism emphasizes that learning occurs most effectively when individuals engage in problem-solving and critical thinking within their ZPD, facilitated by guided interactions with peers or mentors [20]. Therefore, future journal clubs should be uniquely structured in a way that pushes participants to their ZPD in order to maximize the potential for learning to occur.

Once participants arrived in the ZPD, their learning was maximized through scaffolding, where facilitators and knowledgeable peers provided structured support to deepen understanding [18]. Residents described this scaffolding as coming from three sources: faculty facilitators, resident facilitators, and peers with strong backgrounds in research study design. Facilitators’ interventions ranged from suggesting alternative research methodologies to encouraging learners to explore the implications of their study design choices. Through such interventions, facilitators not only helped to fill knowledge gaps but also encouraged learners to engage in deeper, more meaningful analysis of research design decisions. The goal of scaffolding is to achieve deeper and long-lasting learning [21]. Therefore, future iterations of the no-prep journal club should aim to leverage the expertise of faculty and resident facilitators.

Residents also expressed an appreciation for the knowledge of their non-facilitator peers who had strong backgrounds in research, often recalling moments in which they “sat back” and “listened” to their more knowledgeable peers. However, rather than passive learning in the traditional sense, this listening occurred within the context of an environment where residents had already identified their knowledge gaps and engaged in active problem-solving. Having recognized the limits of their understanding, they were primed to absorb new insights from their peers, making this process a form of active knowledge reception rather than passive observation. In this way, the no-prep journal club provided a rich context for learning, where participants alternated between constructing their own understanding and incorporating new information through guided discussion. This dynamic reflects the concept of peer-assisted learning, in which learners at different levels of expertise support one another in constructing knowledge [22]. In these instances, residents with prior experience in study design acted as informal facilitators, offering insights into research methodologies, statistical analyses, and potential biases that might not have been immediately apparent to others. Such peer-led discussions provided additional scaffolding, reinforcing key concepts while allowing participants to engage in active problem-solving and dialogue. By verbalizing their thought processes and explaining research concepts to their colleagues, knowledgeable residents not only reinforced their own understanding but also helped scaffold the learning of their less-experienced peers.

The interactive discussions in the no-prep journal club fostered independence in participants’ ability to analyze research literature, ultimately enhancing residents’ ability to apply study findings to patient care. By actively engaging in discussions that examined study design and methodology in greater depth, residents not only refined their ability to critique research but also became more discerning in how they translated findings into clinical practice. For example, residents mentioned an increased awareness of how primary and secondary outcomes were defined in trials and how that impacted their interpretation of treatment efficacy. This aligns with existing literature on EBM, which emphasizes the importance of critical appraisal skills in clinical decision-making [23]. These findings underscore the importance of journal clubs in their ability to influence patient care by equipping physicians with a more nuanced understanding of research data.

Many residents expressed positive opinions regarding the clinical vignettes presented at the beginning and end of the no-prep journal club. This highlights a problem-centered approach as one of the key principles of andragogy [24]. Learners are most engaged when educational content is immediately applicable to their professional roles in a problem-centered manner [24]. By anchoring research discussions within real-world patient scenarios, the journal club made critical appraisal feel directly relevant to residents' daily clinical practice while also providing a model for applying critical appraisal to their practice. The presence of the clinical vignettes may therefore have been an underlying force behind the reported changes in clinical practice.

### Limitations & transferability

This study has limitations that should be considered when interpreting the findings. The reliance on self-reported data from participants, such as interviews, may introduce the possibility of socially desirable responses or interviewer effects. We chose to mitigate this possibility by having an investigator (SE) with no role or relationship to the residency program conduct the interviews to encourage candid responses. Regarding transferability, these findings may apply to residency programs that institute journal club as part of their didactic curriculum, during either an academic half-day or noon conference format. However, these findings may be less applicable to graduate students, post-doctorate students or research personnel who may have more dedicated time to read research articles and view journal club as a critical component to advancing their skills in research methodology for their own research interests and careers. The researchers’ biases and perspectives may have influenced data collection and interpretation. We have aimed to mitigate this with our reflexivity statement and by engaging in progressive subjectivity during the analysis process [17]. By identifying and articulating our own biases and how these biases shifted throughout the research process, acknowledging subjectivity enabled us to reflect on how our perspectives may have influenced data interpretation. Member checking was another important step, where participants were given the opportunity to review and provide feedback on our findings, ensuring that the interpretations accurately reflected their experiences [16]. We chose a constructivist theoretical framework through which to interpret our data and recognize the possibility that others who choose an alternative theoretical framework may draw conclusions that differ from ours.

### Future directions

Future studies should explore how different facilitation styles influence the effectiveness of the no-prep journal club format. Given the critical role of scaffolding in the learning process, further research could examine how faculty versus peer facilitators shape engagement, knowledge retention, and critical appraisal skills. A potential next step could be a multi-center quantitative study assessing changes in residents’ critical appraisal abilities over time, comparing those who participate in no-prep journal clubs to those who participate in traditional formats. Standardized assessment tools, such as the Fresno Test of Competence in Evidence-Based Medicine, could be used to measure improvements in research literacy and study design evaluation [25]. Another avenue for research is the long-term effect of this format on residents’ motivation to engage with research that will then be helpful for building skills to maintain continuing medical education-relevant habits and lifelong learning, including whether it influences their likelihood of conducting their own studies or integrating EBM more effectively into clinical decision-making. Lastly, while our findings suggest that the no-prep journal club format fosters an engaging and psychologically safe learning environment, future research should explore whether similar active learning approaches could be applied in other medical education settings for resident education, such as interdisciplinary case discussions.

## Conclusions

The no-prep journal club format offers an alternative approach to journal club that prioritizes active learning, collaborative engagement, and psychological safety. By eliminating pre-session preparation requirements, this model fosters an inclusive environment where participants can critically engage with study design, recognize knowledge gaps, and apply findings to clinical practice. Our findings highlight how this format effectively identifies gaps in learners’ knowledge or their ZPDs, in alignment with principles of constructivism and our theoretical framework. The value of facilitator-provided scaffolding and clinical relevance in enhancing the journal club experience also became apparent. While this study provides valuable insights into the benefits of this format, further research is needed to refine and optimize its implementation across diverse residency programs. By continuing to explore innovative approaches to medical education, we can better equip trainees with the skills necessary for critical appraisal, evidence-based practice, and lifelong learning.

## Supplementary Information

Supplementary material 1.

## Data Availability

The data that support the findings of this study are available on request from the corresponding author within a three-year period after the study’s completion. The data are not publicly available due to privacy and ethical restrictions.

## Abbreviations

PGY: Post-graduate year
ZPD: Zone of Proximal Development
EBM: Evidence-based medicine
