# Supplementary material for: Exploring neurology resident experiences with a no-prep journal club to learn research study design and critical appraisal
Source: BMC Med Educ. 2025 Dec 29;25:1732. doi: 10.1186/s12909-025-08275-4 (PMC12750726; doi:10.1186/s12909-025-08275-4)
Supplement: Supplementary file 1 — Supplementary material 1. [file 12909_2025_8275_MOESM1_ESM.docx]

I would like to start by thanking you for making the time to speak with me. As mentioned over email, this interview is part of a research study to better understand how the no-prep journal club format from the 2023-2024 academic year facilitated learning around evidence based and informed practice and how we can better structure educational sessions to improve education of neurology residents on critical appraisal and research methodology.

Your input is valuable and will be used to inform future iterations of the journal club curriculum. Just to confirm, we’d like to keep this interview to about 30 minutes. If you need a break or to stop at any time, please let me know.

During this interview, I’ll ask you a few questions about your experience during the journal club sessions. Please be aware that there are no wrong answers, and we’re doing these interviews to better understand your perspective. We’re interested in hearing specific descriptions about your experience and the more detailed you can be in describing your experiences, the more helpful it will be. We’re conducting these interviews to improve future curriculum, so honest, candid and critical perspectives are highly appreciated.

With your permission, I’d like to record this call. The recording will only be used to help us in our research, and it will not be shared with anyone except those with a need-to-know and who are included as investigators on the IRB. Recording this call will also help me, so I can listen more intently to your responses! Is this okay with you?

Finally, I want to confirm that you’ve received the information sheet as part of this study — is that correct? Have you had a chance to review it? Did you have any questions about it?

Great. Do you have any questions for me before we really start?

Okay, I am going to begin asking you questions about your experience in the no-prep journal club. As a general reminder, the no-prep journal club format is referring to the journal clubs conducted monthly between January and April of 2024 during the Wednesday academic half-day. During this format, you were presented with the background and research question by the resident facilitator and then asked various questions including how review of the article would change your clinical practice. You were also asked to design a study that would best address the research question.

**Interview questions:**

***Background questions***

1. What PGY-year are you?
2. How many journal clubs did you attend?
3. Do you have formal training in research, such as a PhD or master’s degree?

***Key questions***

1. Tell me how this journal club format affected your understanding of research study design.
   1. Describe some examples of challenges during the journal club that opposed learning.
      - 1. Optional prompts:
           1. Could you provide a specific example of this?
           2. Could you provide more details?
   2. Describe some examples of times when you felt your learning was particularly enhanced.
      - 1. Optional prompts:
           1. Could you provide a specific example of this?
           2. Could you provide more details
2. Tell me how this journal club format affected how you apply research results to patient care.
   1. Follow-up prompts: Examples may include specific patients or a framework with which you now read research papers and consider applying them to clinical practice.
   2. Follow-up prompts: Could you please give an example of this? Could you please tell me more about this? Can you tell me more about or explain?
3. Briefly, what knowledge did you have of study design before participating in this journal club?
4. How did you apply that knowledge to this journal club discussion?
   1. Clarification: Different residents have different backgrounds and exposure to research. How did you use your pre-existing knowledge to contribute to discussions?
   2. Follow-up prompts: Could you please give an example of this? Could you please tell me more about this? Can you tell me more about or explain?
5. When reflecting upon how this journal club format differs from other journal clubs you have participated in the past, can you share how this format affected your learning in general?
   1. Follow-up prompts: This could be either positively, negatively, or in another manner.
6. In a prior study, residents expressed difficulty with finding time to prepare for journal club yet acknowledged the importance of preparation. To address this, this year we implemented a no-prep format. How did the lack of preparation affect your experience in the journal club?
7. Is there anything else you would like to add?

***Additional Questions (if there is extra time)***

1. Please describe one take-away you learned after participating in the no-prep journal club curriculum this year.
2. What are your impressions of the decision to open and close the journal club with a clinical case?
3. Describe one behavior that you learned from journal club that you will apply to the critical appraisal of the next article you read.
